# The influence of vulnerability on depression among Japanese university athletes

**DOI:** 10.3389/fspor.2022.1003342

**Published:** 2023-01-12

**Authors:** S. Yamaguchi, Y. Kawata, Y. Murofushi, T. Ota

**Affiliations:** ^1^ Faculty of Health and Sports Science, Juntendo University, Inzaishi, Chiba, Japan; ^2^ Graduate School of Health and Sports Science, Juntendo University, Inzaishi, Chiba, Japan; ^3^ Institute of Health and Sports Science and Medicine, Juntendo University, Inzaishi, Chiba, Japan

**Keywords:** depression, Japanese university athletes, vulnerability, mental health, structural equation modeling

## Abstract

**Objective:**

This study examined the estimated causal relationship between vulnerability and depressive symptoms in Japanese university athletes and how the degree of vulnerability affects depressive symptoms.

**Materials and methods:**

In Study 1, 248 Japanese university athletes completed a continual survey from Time 1 to Time 3. In Study 2, 562 Japanese university athletes responded to another survey during the same period. Structural equation modeling was performed to estimate the causal relationship using the cross-lagged effects model for the three waves. Next, a binomial logistic regression analysis was performed to examine the influence of vulnerability on depression.

**Results:**

Results of the cross-lagged effects model showed that all paths from vulnerability to depressive symptoms were significant, and all paths from depressive symptoms to vulnerability were not significant. Thus, vulnerability was the causative variable and depressive symptoms were the outcome variables within the causal relationship. The logistic regression results showed that those with high vulnerability were 1.7 times more likely to have moderate or higher depressive symptoms than those with low vulnerability. Vulnerable individuals are at a higher risk for developing depressive symptoms. By verifying the causal relationship between vulnerability and depressive symptoms, we can contribute to the enhancement of mental health care in accordance with the weakest link model. Appropriate psychological support for athletes can decrease depression and improve their mental health.

## 1. Introduction

Exercise and physical activity have a positive effect on mental health (1); however, this is not always the case for athletes, as there are instances in which stress factors distinctive to a competitive life impair athletes' mental health. Fatigue from training has also been shown to lead to mental health problems, such as depressive symptoms (2), burnout, eating disorders, and, in severe cases, suicide (3, 4). Depression is one of the leading causes of non-fatal health loss worldwide (5). The prevalence of depression among college athletes globally has been found to range from 15.6% to 21.0% (6), with several top athletes having reported that they suffer from serious depression (7). Thus, it is highly possible that the incidence of mental health problems, their severity, and the risk of developing symptoms have become serious challenges for athletes (8). However, not all athletes develop mental health problems. Some athletes can respond flexibly when faced with stressors (9). Conversely, people with a negative predisposition, such as those with a higher level of vulnerability, are more likely to display maladaptation when faced with a stressful event than those with a more positive predisposition (10).

Several models of cognitive vulnerability to depression have been presented. Abela and Sarin (11) proposed the weakest link hypothesis, which examines the influence of multiple measures of cognitive vulnerability, postulating that a person's most maladaptive score on a set of indices is the best predictor of risk for depression. Individuals' overall propensity to depression may lie solely with their most vulnerable cognitive characteristics. Thus, indices of a person's average vulnerability may be relevant in predicting depression. Yamaguchi et al. (12) developed the weakest link model, focusing on athletes' vulnerability to explain the development of mental health problems among them. Vulnerability refers to a cognitive structure that is susceptible to stress (13) and is defined as “a susceptibility to damaging oneself, a possible state of brittle or emotional hurt” (14). According to this model, athletes with high levels of vulnerability may suffer damage to their mental health if they encounter an injury factor, which may worsen their competitive performance or even lead to a mental health disorder (12). In fact, vulnerable individuals are more likely to develop negative interpretations of an event when faced with stress factors, resulting in the development of depression (15, 16). Furthermore, people with a negative predisposition, for example, those with a higher level of vulnerability, are more likely to display maladaptation when faced with a stressful event than those with a more positive predisposition (10). Therefore, athletes’ level of vulnerability can affect their level of psychological distress and pain.

Studies on vulnerability showed that women score significantly higher on vulnerability than men, indicating that they are more vulnerable (17). It has also been established that, compared to other students, freshmen are more vulnerable (18). In addition, a relationship between vulnerability and mental health (General Health Questionnaire) and depressive symptoms (self-rating depression scale) has been reported (12, 17). Moreover, positive correlations have been confirmed between stress vulnerability, anxiety symptoms, and depression symptoms in medical and humanities students (19). Furthermore, Satici (20) reported that vulnerability has a negative path of hope and well-being, and that high vulnerability leads to low wishful thinking and contributes to a decline in happiness. Therefore, the higher the vulnerability, the more the person's mental health would be impaired, and more depressive symptoms would be displayed. Since vulnerability is related to mental health symptoms, it is important to focus on an individual's vulnerability to prevent mental health disorders related thereto.

Research on this topic, thus far, has been limited to cross-sectional studies. Previous studies have clarified the correlation between factors related to vulnerability, but they have been unable to grasp the temporal precedence between these variables. Therefore, it has not yet been clarified whether depressive symptoms increase due to high vulnerability, or whether increased depressive symptoms lead to higher vulnerability. For example, when an athlete faces a stress factor, the athlete may be negatively affected by it and exhibit depressive symptoms. Conversely, when a stress factor is present, the athlete's depressive symptoms may increase, leading to further negative effects. The exact mechanism remains unclear. Cognitive theories of depression define vulnerability as an internal and stable feature of individuals that predisposes them to develop depression following the occurrence of negative events (21). According to Abela and Hankin (22), the higher an individual's level of cognitive vulnerability, the less stressful the negative event need to be to trigger the onset of depressive symptoms or episodes. Cognitive theorists have conducted considerable research on the relationship between vulnerability and depressive symptoms (23–25); however, no study has been conducted on “vulnerability” alone. In addition, research on vulnerability factors for athletes has not been conducted. Examining the causal relationship between vulnerability *per se* and depressive symptoms in athletes may contribute to the cognitive theory of vulnerability.

One of the methods for examining this relationship is an analytical approach, using a cross-lagged effects model (cross-lagged panel design), which is a statistical method that uses longitudinal data and analyzes a causal relationship after incorporating the possibility that two variables might affect each other in both directions (26). Previous studies on the causal relationship between vulnerability and depressive symptoms have assumed that depressive symptoms (stress response) were outcomes. However, it is important to clarify the causal relationship between the two variables using a cross-lagged effects model to confirm whether this assumption is correct. If we assume that depressive symptoms are the outcome variable caused by vulnerability, the relationship wherein “the higher the vulnerability, the more the depressive symptoms are exhibited,” can be estimated. At this point, logistic regression analysis is considered useful as a test for examining the degree of depressive symptoms caused by vulnerability. Logistic regression analysis is a model used to predict probabilities by linking two-valued variables that represent the occurrence of events with multiple factors. As an example of analytical use, it is a statistical method widely used in various scientific fields (e.g., systems engineering and medicine) to examine, for example, the occurrence of diseases and the presence or absence of earthquakes. By using logistic regression analysis for the variables examined in this study, it would be possible to estimate the degree to which depressive symptoms are affected by an individual's vulnerability.

Previous studies have clarified the strength of the correlation between vulnerability and depressive symptoms (12, 27). A study conducted in the field of biological psychiatry reported that chronic social stress increases vulnerability (28). According to Ingram and Luxton (15), the route from vulnerability to disorder is explained by various versions of the vulnerability-stress model, as the way in which interactions between predisposing factors (vulnerabilities) and stress influence the development of a disorder. Furthermore, Zuroff et al. (29) inquired “whether vulnerable individuals merely describe themselves as being more self-critical or dependent, or whether they become more self-critical or dependent when they experience depressive symptoms.” Therefore, the stressor is expected to increase an individual's vulnerability and, consequently, cause a stress response. However, while several studies have examined the relationship between vulnerability and stress responses, the aspect of causation is ignored. In fact, when considering the extent to which a vulnerable state affects stress responses, researchers regarded the stress response as the dependent variable, and vulnerability was viewed as an intervening factor between the stressor and stress response. Thus, we believe that clarifying the temporal and causal relationships between these two variables can provide a novel perspective regarding the weakest link model and possibly help in developing an approach to provide increased early support to vulnerable people. If the cause-and-effect relationship—such as “when people are emotionally hurt, they experience increased depressive symptoms”—is identified, it will be possible to estimate the risk of developing depression, which will provide an opportunity to prevent mental health problems.

### 1.1. Purpose

To examine the effect of vulnerability on depressive symptoms, this study was divided into two parts—Study 1 aimed to elucidate the causal relationship between vulnerability and depressive symptoms, and Study 2 sought to examine the effect of vulnerability on depressive symptoms. The hypothesis of Study 1 is as follows:

H1: There is a causal relationship between vulnerability and depressive symptoms, of which depressive symptoms are the outcome.

The purpose of Study 2 was to examine the extent to which the degree of vulnerability affects depressive symptoms, using logistic regression. The hypothesis of Study 2 is as follows:

H2: The higher the score for vulnerability, the higher the risk of depressive symptoms occurring.

## 2. Study 1

### 2.1. Survey period

The survey period was divided into Time 1 (from mid-April to mid-May 2018), Time 2 (from late June to early July 2018), and Time 3 (October 2018).

### 2.2. Participants

A total of 248 university athletes (161 men and 87 women; average age = 19.0 years, *SD* = 0.85), completed the entire survey from Time 1 to Time 3. The participants were student athletes from university sports teams or sports clubs, who participated in competitions in the Kanto areas of Japan. Participants from 22 sports—including track and field, soccer, baseball, and basketball—who competed at international, national, and regional levels were included in this study. Athletes who participated for leisure or refreshment purposes were not included. Athletes who had developed injuries or illnesses or had left the club during the research period were excluded, as were staff. It is reported that the desirable sample size for model verification is 200 samples or more (30). In addition, although the sample size was 143 in the study using the cross-lagged model (31), the sample size in our study was set to about 200 in consideration of missing responses and non-participation in the longitudinal survey.

### 2.3. Method

The survey was administered through the group survey method, using a questionnaire. Once students had received the questionnaire, read the section related to ethical considerations on the cover page, and agreed to participate, they proceeded to respond to the questions. The completed questionnaires were collected immediately.

### 2.4. Ethical considerations

Before starting the survey, the participants were fully informed verbally and in writing that participation in the survey was voluntary, that they would not be disadvantaged if they did not participate, and that the results would be used solely for the purpose of this study. Participants provided informed consent prior to the study. This study was conducted with the approval of the institutional review board of the institution to which the first author belongs (No. 30-103).

### 2.5. Investigation

The contents of the questionnaire survey were as follows:

#### 2.5.1. Basic attributes

As demographic data, we collected participant information regarding their gender, age, grade, and the sport types they participated in.

#### 2.5.2. Athletic vulnerability scale

This scale was developed by Yamaguchi et al. (17) to measure athletes' vulnerability. It consists of 12 items with three factors: vulnerability toward low interpersonal evaluation, vulnerability toward unstable performance, and vulnerability toward denial or being ignored by others. Participants were to respond using a rating scale from 1 (“I completely disagree”) to 4 (“I completely agree”). In this scale, the higher the score of vulnerability (easier to hurt the mind), the more likely it is to lead to mental health problems. The average score of all items was taken as the respondent's score on this scale.

#### 2.5.3. Depression affinity self-evaluation scale

Zung's Self-Rating Depression Scale (32) was used to measure depressive symptoms. Participants responded on a four-point scale from 1 (“some of the time”) to 4 (“most of the time”); the higher the score, the more likely the person is to be depressed.

### 2.6. Analytical method

An analysis of variance with correspondence was used to determine how vulnerability and depressive symptoms change over time, based on measurements at three-time points. Next, a causal relationship was estimated, using a three-wave cross-delay-effect model. We specifically verified the cross-lagged effects model for three waves (Time 1, Time 2, and Time 3), using structural equation modeling. The comparative-fit index (CFI), goodness-of-fit index (GFI), adjusted goodness-of-fit index (AGFI), and root-mean-square error of approximation (RMSEA) were used as indicators of the goodness of fit of the model. We used IBM's SPSS Statistics [IBM SPSS Statistics for Windows, version 28 (IBM Corp., Armonk, NY, United States)] and the Amos 28 software programs for data analyses.

### 2.7. Results

#### 2.7.1. The causal relationship between vulnerability and depressive symptoms

The analysis of variance showed that vulnerabilities and depressive symptoms fluctuate over time. Vulnerability increased more during Time 3 than during Time 1 or Time 2. Depressive symptoms were higher in Time 1 than in Times 2 or 3 (Table 1).

**Table 1:** Time-series basic data and results for vulnerability and depressive symptoms.

	Time 1	Time 2	Time 3	*F*-value	*p*-value	Multiple comparison
Vulnerability	2.6	2.6	2.7	10.47	.001***	1 < 3, *p* < .01 2 < 3, *p* < .001
Depression	40.7	40.5	39.3	4.23	.05*	1 > 3, *p* < .05 2 > 3, *p* < .01

* *p* < .05, ***p < .001.

**Figure 1:**
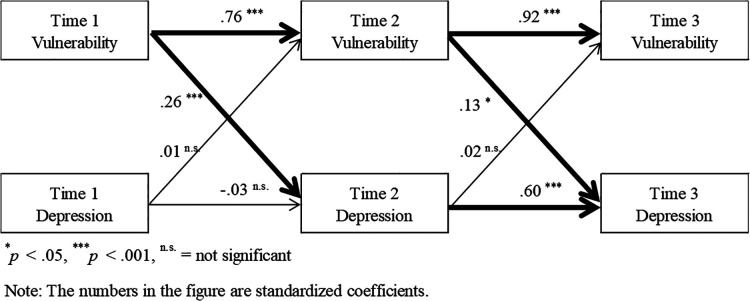
Estimated results of a causal relationship between vulnerability and depressive symptoms.

Analysis by the cross-lagged effects model showed a significant relationship in the path of vulnerability in Time 1 to depressive symptoms, and vulnerability in Time 2 to depressive symptoms, showing a significant relationship between depressive symptoms and vulnerability in Time 1 (Figure 1). There was no significant difference between Time 2 and Time 3 for vulnerability and depressive symptoms. The model's goodness of fit was as follows: CFI = .98, GFI = .98, AGFI = .93, and RMSEA = .07.

## 3. Study 2

### 3.1. Survey period

The survey period was October 2018, the same as Time 3 in Study 1.

### 3.2. Participants

The participants were 562 athletes (376 men and 186 women; average age = 20.1 years, *SD* = 1.30), who belonged to a competitive athletic club or university in the Kanto area. Participants from Study 1 were not included. Participants from 21 individual sports and team sports—such as track and field, gymnastics, and soccer—who competed at international, national, local, prefectural, and district levels were included in this study.

### 3.3. Survey method

The survey method was the same as that for Study 1.

### 3.4. Ethical considerations

The ethical considerations were the same as those in Study 1.

### 3.5. Investigation

The questionnaires used were the same as those used for Study 1.

### 3.6. Analytical method

We investigated the effect of vulnerability on depressive symptoms. First, the descriptive statistics for each variable were calculated, and t-tests and analysis of variance were performed to clarify the confounding factors obtained from the demographic data. Subsequently, the degree of influence (odds ratio) on depressive symptoms was examined, based on the vulnerability score. The vulnerability score was classified as a median, two-valued variable (high group/low group), and taken as the independent variable, and the depressive symptom score was classified by the cutoff value of 47 points (33), which is considered as “moderately depressed.” A binomial logistic regression analysis was performed with the two-valued variable as the dependent variable, and the confounding factors as the covariate. We used IBM's SPSS Statistics [IBM SPSS Statistics for Windows, version 28 (IBM Corp., Armonk, NY, United States)] software program for data analyses.

### 3.7. Results

#### 3.7.1. Effects of vulnerability on depressive symptoms

The analysis revealed gender and age as confounding factors; women scored significantly higher on the vulnerability scale than men, and older people were shown to be more vulnerable than younger people. Based on these findings, the abovementioned confounding factors were captured as covariates in the logistic regression analysis.

The logistic regression analysis showed that when the low vulnerability group was 1.0, the high vulnerability group had a 1.7-fold increased risk of moderate or higher depression (Exp *β* = 1.70, 95% confidence interval = 1.02–2.83, *p* < .05; Table 2).

**Table 2:** Results of the effects of vulnerability on depressive symptoms.

	*β*	Odds ratio	*p*-value	95% Confidence intervals	
Vulnerability	.522	1.704	.039*	1.027	2.825
Gender	.103	1.109	.704	0.651	1.890
Age	.002	1.002	.659	0.995	1.008
	−2.286	.102	.001		

* *p* < .05.

## 4. Discussion

The purpose of this study was to explore the causal relationship between vulnerability and depressive symptoms in Japanese university athletes, and to examine the effect of high vulnerability on depressive symptoms.

### 4.1. The causal relationship between vulnerability and depressive symptoms in Japanese university athletes

The cross-delayed effect model in Study 1 revealed that all paths from vulnerability to depressive symptoms were significant, and that all paths from depressive symptoms to vulnerability were not significant. Accordingly, it was possible to estimate the causal relationship between these variables. However, caution is needed with respect to the model's goodness of fit indices, which were.90 or higher for the CFI, GFI, and AGFI, and.07 for the RMSEA (34). While an RMSEA ≤ .06 is considered acceptable (35), an RMSEA ≥ .10 is not (36). Based on these criteria, although the CFI, GFI, and AGFI were within the reference values, the RMSEA was not sufficiently good. Therefore, increasing the number of participants and including more error correlations could improve the model's fit. However, we believe that such actions are arbitrary and not highly desirable. Based on Shi et al.'s (37) assertion that the model size has an important impact on the population values of CFI and RMSEA, in the present study, we postulated that increasing the number of participants would stabilize the fit of the model. Indeed, we found that the model size (*p*) significantly affected the population values of the CFI, TLI, and RMSEA. Even if the model fit exceeds the standard value, it may be possible to bring about a fit that falls within the standard value by increasing the sample size. Hence, it would be desirable to conduct a new survey instead of increasing the sample size arbitrarily. Therefore, based on this study's results, further investigation is recommended in future research. Regarding the estimation of causality using the cross-delayed effect model, no significant difference was found in any of the paths from depressive symptoms to vulnerability. Thus, the manifestation of depressive symptoms escalates when vulnerability increases. Previous studies dealing with vulnerability and depressive symptoms have treated depressive symptoms as outcomes due to vulnerability (12, 17, 33). From this, it has been reported that there is a positive relationship between vulnerability and mental health and depressive symptoms, and that the more vulnerable the person, the poorer the mental health. However, although causal relationships have not been estimated in those studies, more conclusive results were obtained in the current study by estimating the causal relationships. Vulnerable individuals display a negative interpretation of life events only when they are confronted with certain stressors, which places them at high risk for depression and other diverse negative outcomes (15). Moreover, when confronted with a new situation, a vulnerable person's first thought might be that they will fail at a newly learned task or they should avoid a new acquaintance (38). From this, it can be inferred that vulnerable people perceive events in a negative light, causing them to feel extra hurt, and as a result of that reaction, it leads to depressive symptoms and poor mental health.

### 4.2. Effects of the degree of vulnerability on depressive symptoms

The logistic regression analysis was performed in Study 2, which showed that those with high vulnerability were 1.7 times more likely to develop depressive symptoms than those with low vulnerability. These facts support the hypothesis of this study: “the higher the score of vulnerability, the higher the risk of depressive symptoms.” Based on the above results, we will subsequently discuss the causal relationship between vulnerability and depressive symptoms, and the effect of vulnerability on depressive symptoms. Regarding the effect of vulnerability on depressive symptoms, depressive symptoms were estimated as an outcome, caused by vulnerability in the previous cross-delayed effects model. Examining how different degrees of vulnerability affect depressive symptoms revealed that the group with high vulnerability was 1.7 times more likely to have depressive symptoms than the group with low vulnerability. In other words, those with high vulnerability were approximately twice as likely to develop depressive symptoms than those who were less vulnerable; this indicates that people with high vulnerability were at a higher risk of developing depression. Xiao et al. (39) evaluated vulnerabilities and depressive symptoms in Chinese university students using a hierarchical linear model analysis. Their results support the findings of this study, as it showed that those with high vulnerability scores have significantly more depressive symptoms than those with low vulnerability scores. Higher weakest link scores would be associated with greater increases in depressive symptoms following negative events (11, 40). According to Abela and Sarin (11), this provides support for operationalizing cognitive vulnerability to depression by utilizing a weakest link approach.

### 4.3. Vulnerability and depressive symptoms according to the weakest link model

Focusing on vulnerability, it is predicted that the degree of subsequent depressive symptoms will differ, depending on the degree of individual vulnerability. In fact, the degree of vulnerability differed from the results of the logistic regression analysis. Given these facts, we could demonstrate the psychology-based “weakest link model” proposed by Yamaguchi et al. (12). Originally, the weakest link model was outlined in engineering as follows: “when a chain is pulled, it begins to break at the weakest part of the chain and gradually collapses” (41, 42). Therefore, it shows that vulnerable people's mental health, which is initially good, is gradually damaged when their weak and fragile parts are attacked. For instance, if adolescents experience hurt as threatening, they recall it repeatedly, which leads to increased stress (43). This is exactly what the weakest link represents. Previous studies have examined the link between vulnerability and all stress responses. However, in most of those studies, researchers judged that vulnerability was the causative variable and stress response was the outcome. Since the path coefficient was significant and the model's goodness of fit was within the permissible range, as previously shown, causality could be inferred; it was appropriate to attribute causality to vulnerability, with depressive symptoms as the outcome. In other words, since vulnerability was the cause and depressive symptoms were the result, it was consequently suggested that vulnerability is associated with depressive symptoms, and that the higher the vulnerability score, the stronger its effect on depressive symptoms. From this, the weakest link model was proven, and it was found that when there is an event in which a person can easily be hurt, the person's mental health is more and more damaged from that incident. In addition, according to Abela and Sarin (11) who advocated the weakest link approach, their study revealed that participants' “weakest links” interacted with the occurrence of negative events to predict increases in depressive symptoms. This study has shown that vulnerability is the causative variable; therefore, we think that further research based on the weakest link will be necessary in the future.

Yamaguchi et al. (44) also examined the impact of mental health as an outcome of vulnerability and suggested that those with high vulnerability are approximately twice as likely to experience deteriorated mental health compared to those with low vulnerability. Therefore, it is conceivable that vulnerable people are more likely to experience poorer mental health. In terms of depressive symptoms and mental health, people who are easily hurt have poorer mental health, implying that early intervention for vulnerable individuals is important. For example, having a social network and team support is an effective way to prevent depression in college athletes (45). Amemiya and Sakairi (31) noted that teammates' support is important for athletes. When interpersonal problems arise due to excessive competition within the team, athletes are unable to receive sufficient support from their teammates, which functions as a stressor for interpersonal exhaustion. Furthermore, the persistence of such symptoms may cause more serious psychological problems (31). These findings demonstrate the importance of a functioning support system for athletes who are particularly vulnerable. According to Yamaguchi et al. (9), vulnerable athletes need to be provided with appropriate emotional support to cope with stressful situations, as they rely heavily on a stress management strategy for emotional regulation. For this reason, it is important to provide emotional and teammate-related support for vulnerable athletes. However, in competitive sports, wherein high performance is imperative, the susceptibility of individuals and the need for support may be perceived as “individual weakness.” It has also been pointed out that excessive stress may lead to burnout and mental health problems (46). In both cases, a preventive approach is important. Hence, the findings of this study imply the need to prevent the development or progression of depressive symptoms in athletes, and this can be used to emphasize the promotion of support for maintaining good mental health. We think that it is important to understand mental health by considering a person's weakest link.

### 4.4. Limitations

This study has two limitations. The first concerns the timing of the survey. The survey was conducted at intervals of three months—that is, in April, July, and October. When conducting a longitudinal survey, it is preferable to have an interval of two to three months. However, the month of April, in which the survey was first conducted, was the beginning of the new semester—a period of excessive vulnerability for some students. Regarding depressive symptoms, a significant difference was observed from Time 2 to Time 3. Therefore, it is possible that experiences of depressive symptoms are more likely to occur during autumn than in spring. Thus, setting the survey timing appropriately is seemingly important and must be taken into consideration.

The second limitation is the absence of stress responses other than depressive symptoms. In this study, depressive symptoms were defined as outcomes. However, psychological variables other than depressive symptoms that adversely affect mental health were predicted. In fact, Yamaguchi et al. (44) conducted research using mental health as an outcome and obtained similar results. Examining factors associated with depressive symptoms and other stress responses and vulnerability may help vulnerable athletes develop coping strategies for maintaining good mental health. Concomitantly, caution must be exercised when interpreting causality. We used a cross-delay effect model to try to estimate the causal relationship between vulnerability and depressive symptoms this time. A causal relationship was estimated only with the obtained data. Although the cause and effect relationship—that people who are easily hurt daily are more likely to develop depressive symptoms—has been confirmed, it is necessary to conduct interview surveys and examine the pathway from hurt to depression. However, further careful verification is necessary in the future.

Additionally, we included athletes who compete at various levels, ranging from international to local. Although an examination of the confounding factors in Study 1 did not reveal a significant difference, the degree of depressive symptoms may differ depending on the competition level. Therefore, future research should consider the differences between individual and group competitions, as well as those between each competition level.

Based on the above, we can expect the development of research that will focus on the prevention, maintenance, and improvement of athletes’ mental health by considering and analyzing the issues listed above, as well as developing strategies to prevent depression.

## 5. Conclusion

In this study, we aimed to explore the causal relationship between vulnerability and depressive symptoms, and examine the effect of high vulnerability on depressive symptoms among Japanese university athletes. The results showed that all paths from vulnerability to depressive symptoms were significant, indicating that vulnerability is a causal factor for depression. Additionally, those with high vulnerability were 1.7 times more at risk of depressive symptoms by than those with low vulnerability. From the sport psychology perspective, understanding athletes' degree of vulnerability is important to prevent adverse effects on their mental health. This is expected to stimulate the development of research focused on preventing mental health problems, as well as maintaining and improving mental health. In the future, it will be necessary to develop mental health support for vulnerable people according to the weakest link model. In addition, regarding the fact—which was verified in this study—that depressive symptoms increase when one is emotionally hurt, it is important to conduct an interview survey to understand the pathway from emotional hurt to depressive symptoms.

## Data Availability

The original contributions presented in the study are included in the article/Supplementary Material, further inquiries can be directed to the corresponding author/s.

## References

[1] Hamer M Stamatakis E Steptoe A. Dose-response relationship between physical activity and mental health: the Scottish health survey. Br J Sports Med. (2009) 43:1111–4. 10.1136/bjsm.2008.046243 18403415

[2] Cadegiani F. Overtraining syndrome in athletes. Cham: Springer (2020).

[3] Peluso MAM de Andrade LHSG. Physical activity and mental health: the association between exercise and mood. Clinics. (2005) 60:61–70. 10.1590/s1807-59322005000100012 15838583

[4] Sundgot-Borgen J Torstveit MK. Prevalence of eating disorders in elite athletes is higher than in the general population. Clin J Sport Med. (2004) 14:25–32. 10.1097/00042752-200401000-00005 14712163

[5] James SL Abate D Abate KH Abay SM Abbafati C Abbasi N Global, regional, and national incidence, prevalence, and years lived with disability for 354 diseases and injuries for 195 countries and territories, 1990–2017: a systematic analysis for the global burden of disease study 2017. Lancet. (2018) 392:1789–858. 10.1016/S0140-6736(18)32279-7 30496104 PMC6227754

[6] Proctor SL Boan-Lenzo C. Prevalence of depressive symptoms in male intercollegiate student-athletes and nonathletes. J Clin Sport Psychol. (2010) 4:204–20. 10.1123/jcsp.4.3.204

[7] Baum AL. Suicide in athletes: a review and commentary. Clin Sports Med. (2005) 24:853–69. 10.1016/j.csm.2005.06.006 16169450

[8] Tahtinen RE Shelley J Morris R. Gaining perspectives: a scoping review of research assessing depressive symptoms in athletes. Psychol Sport Exerc. (2021) 54:101905. 10.1016/j.psychsport.2021.101905

[9] Yamaguchi S Kawata Y Murofushi Y Shibata N Ota T. Psychological vulnerability associated with stress coping strategies in Japanese University athletes. J Clin Sport Psychol. (2022) 1(aop):1–15. 10.1123/jcsp.2021-0084

[10] Ishizu K Ambo H. Vulnerability factors for school stress among junior high school students: over-adaptation and evaluation of emotion. Japanese J Educ Psychol. (2013) 84(2):130–7. 10.4992/jjpsy.84.130 23848000

[11] Abela JR Sarin S. Cognitive vulnerability to hopelessness depression: a chain is only as strong as its weakest link. Cognit Ther Res. (2002) 26(6):811–29. 10.1023/A:1021245618183

[12] Yamaguchi S Kawata Y Kaneko Y Nakamura M Shibata N Hirosawa M. Relationship between vulnerability and depression among Japanese University athletes. Juntendo Med J. (2018) 64(1):60–3. 10.14789/jmj.2018.64.JMJ18-P31

[13] Sinclair VG Wallston KA. The development and validation of the psychological vulnerability scale. Cognit Ther Res. (1999) 23(2):119–29. 10.1023/A:1018770926615

[14] Hayashi K. A study of vulnerability (Kizutsukiyasusa Nitsuiteno Ichikousatsu). Mem Shiraume Gakuen Coll. (2002) 38:1–10 (In Japanese).

[15] Ingram RE Luxton DD. Vulnerability-stress models. In: Hankin BL Abela JRZ, editors. Development of psychopathology: A vulnerability-stress perspective. Thousand Oaks, CA: Sage Publications (2005). p. 32–46.

[16] Monroe SM Simons AD. Diathesis-stress theories in the context of life stress research: implications for the depressive disorders. Psychol Bull. (1991) 110:406–25. 10.1037/0033-2909.110.3.406 1758917

[17] Yamaguchi S Kawata Y Nakamura M Hirosawa M Shibata N. Development of the athletic vulnerability scale: an examination of vulnerability among university athletes and related factors. Juntendo Med J. (2019) 65:136–48. 10.14789/jmj.2019.65.JMJ18-OA14

[18] Nogueira MJ Sequeira C Sampaio F. Gender differences in mental health, academic life satisfaction and psychological vulnerability in a sample of college freshmen: a cross-sectional study. J Gend Stud. (2021) 31(8):1–10. 10.1080/09589236.2021.1979945

[19] Bunevicius A Katkute A Bunevicius R. Symptoms of anxiety and depression in medical students and in humanities students: relationship with big-five personality dimensions and vulnerability to stress. Int J Soc Psychiatry. (2008) 54(6):494–501. 10.1177/0020764008090843 18974188

[20] Satici SA. Psychological vulnerability, resilience, and subjective well-being: the mediating role of hope. Pers Individ Differ. (2016) 102:68–73. 10.1016/j.paid.2016.06.057

[21] Ingram RE Miranda J Segal ZV. Cognitive vulnerability to depression. New York: Guilford Press (1998).

[22] Abela JR Hankin BL. Cognitive vulnerability to depression in children and adolescents: A developmental psychopathology perspective. New York: The Guilford Press (2008).

[23] Abramson LY Seligman MEP Teasdale J. Learned helplessness in humans: critique and reformulation. J Abnorm Psychol. (1978) 87:49–74. 10.1037/0021-843X.87.1.49 649856

[24] Beck AT. Depression: Clinical, experimental, and theoretical aspects. New York: Harper & Row (1967).

[25] Blatt SJ Zuroff DC. Interpersonal relatedness and self-definition: two prototypes for depression. Clin Psychol Rev. (1992) 12:527–62. 10.1016/0272-7358(92)90070-O

[26] Finkel SE. Causal analysis with panel data. Thousand Oaks, CA: Sage Publications (1995).

[27] Hankin BL Abramson LY. Measuring cognitive vulnerability to depression in adolescence: reliability, validity, and gender differences. J Clin Child Adolesc Psychol. (2002) 31(4):491–504. 10.1207/S15374424JCCP3104_8 12402568

[28] Zhang Y Lu W Wang Z Zhang R Xie Y Guo S Reduced neuronal cAMP in the nucleus accumbens damages blood-brain barrier integrity and promotes stress vulnerability. Biol Psychiatry. (2020) 87(6):526–37. 10.1016/j.biopsych.2019.09.027 31812254

[29] Zuroff DC Mongrain M Santor DA. Conceptualizing and measuring personality vulnerability to depression: comment on Coyne and Whiffen (1995). Psychol Bull. (2004) 130(3):489–511. 10.1037/0033-2909.130.3.489 15122935

[30] Iacobucci D. Structural equations modeling: fit indices, sample size, and advanced topics. J Consum Psychol. (2010) 20(1):90–8. 10.1016/j.jcps.2009.09.003

[31] Amemiya R Sakairi Y. Examining the relationship between depression and the progression of burnout among Japanese athletes 1, 2. Jpn Psychol Res. (2022) 64(4):373–84. 10.1111/jpr.12332

[32] Zung WWK. A self-rating depression scale. Arch Gen Psychiatry. (1965) 12:63–70. 10.1001/archpsyc.1965.01720310065008 14221692

[33] Zung WWK. The depression status inventory: an adjunct to the self-rating depression scale. J Clin Psychol. (1972) 28(4):539–43. 10.1002/1097-4679(197210)28:4<539::AID-JCLP2270280427>3.0.CO;2-S 5080837

[34] Toyoda H. Covariance structure analysis (amons version). Tokyo: Tokyo Books (2007).

[35] Hu LT Bentler PM. Cutoff criteria for fit indexes in covariance structure analysis: conventional criteria versus new alternatives. Struct Equ Modeling. (1999) 6(1):1–55. 10.1080/10705519909540118

[36] Browne MW Cudeck R. Alternative ways of assessing model fit. In: Bolen KA Long JS, editors. Testing structural equation models. Newbury Park, CA: Sage (1993). p. 136–62.

[37] Shi D Lee T Maydeu-Olivares A. Understanding the model size effect on SEM fit indices. Educ Psychol Meas. (2019) 79(2):310–34. 10.1177/0013164418783 30911195 PMC6425088

[38] Beevers CG. Cognitive vulnerability to depression: a dual process model. Clin Psychol Rev. (2005) 25(7):975–1002. 10.1016/j.cpr.2005.03.003 15905008

[39] Xiao J Qiu Y He Y Cui L Auerbach RP McWhinnie CM “Weakest link” as a cognitive vulnerability within the hopelessness theory of depression in Chinese university students. Stress Health. (2016) 32:20–7. 10.1002/smi.2571 24639362 PMC4379125

[40] Abela JR McGirr A. Operationalizing cognitive vulnerability and stress from the perspective of the hopelessness theory: a multi-wave longitudinal study of children of affectively ill parents. Br J Clin Psychol. (2007) 46(4):377–95. 10.1348/014466507X192023 17535532

[41] Weibull W. A statistical distribution function of wide applicability. J Appl Mech. (1951) 18(3):293–7. 10.1115/1.4010337

[42] Weibull W. A statistical theory of strength of materials. Generalstabens Litografiska Anstalts Förlag, Stockholm. Mod Mech Eng. (1939) 6(4).

[43] Joseph S Williams R. Understanding posttraumatic stress: theory, reflections, context and future. Behav Cogn Psychother. (2005) 33:423–41. 10.1017/S1352465805002328

[44] Yamaguchi S Kawata Y Noguri R Murofushi Y Ota T. Effect of vulnerability on mental health among university athletes. Jpn J Appl Psychol. (2022) 65:136–48. 10.24651/oushinken.47.3_209

[45] Armstrong S Oomen-Early J. Social connectedness, self-esteem, and depression symptomatology among collegiate athletes versus nonathletes. J Am Coll Health. (2009) 57:521–6. 10.3200/JACH.57.5.521-526 19254893

[46] Hägglund K Kenttä G Thelwell R Wagstaff CR. Is there an upside of vulnerability in sport? A mindfulness approach applied in the pursuit of psychological strength. J Sport Psychol Action. (2019) 10(4):220–6. 10.1080/21520704.2018.1549642

